# Neighborhood Socioeconomic Circumstances and the Co-Occurrence of Unhealthy Lifestyles: Evidence from 206,457 Australians in the 45 and Up Study

**DOI:** 10.1371/journal.pone.0072643

**Published:** 2013-08-15

**Authors:** Xiaoqi Feng, Thomas Astell-Burt

**Affiliations:** 1 Centre for Health Research, School of Medicine, University of Western Sydney, Sydney, Australia; 2 School of Science and Health, University of Western Sydney, Sydney, Australia; Hunter College, City University of New York (CUNY), CUNY School of Public Health, United States of America

## Abstract

**Background:**

Research on the co-occurrence of unhealthy lifestyles has tended to focus mainly upon the demographic and socioeconomic characteristics of individuals. This study investigated the relevance of neighborhood socioeconomic circumstance for multiple unhealthy lifestyles.

**Method:**

An unhealthy lifestyle index was constructed for 206,457 participants in the 45 and Up Study (2006–2009) by summing binary responses on smoking, alcohol, physical activity and five diet-related variables. Higher scores indicated the co-occurrence of unhealthy lifestyles. Association with self-rated health, quality of life; and risk of psychological distress was investigated using multilevel logistic regression. Association between the unhealthy lifestyle index with neighborhood characteristics (local affluence and geographic remoteness) were assessed using multilevel linear regression, adjusting for individual-level characteristics.

**Results:**

Nearly 50% of the sample reported 3 or 4 unhealthy lifestyles. Only 1.5% reported zero unhealthy lifestyles and 0.2% had all eight. Compared to people who scored zero, those who scored 8 (the ‘unhealthiest’ group) were 7 times more likely to rate their health as poor (95%CI 3.6, 13.7), 5 times more likely to report poor quality of life (95%CI 2.6, 10.1), and had a 2.6 times greater risk of psychological distress (95%CI 1.8, 3.7). Higher scores among men decreased with age, whereas a parabolic distribution was observed among women. Neighborhood affluence was independently associated with lower scores on the unhealthy lifestyle index. People on high incomes scored higher on the unhealthy lifestyle index if they were in poorer neighborhoods, while those on low incomes had fewer unhealthy lifestyles if living in more affluent areas.

**Interpretation:**

Residents of deprived neighborhoods tend to report more unhealthy lifestyles than their peers in affluent areas, regardless of their individual demographic and socioeconomic characteristics. Future research should investigate the trade-offs of population-level versus geographically targeted multiple lifestyle interventions.

## Introduction

To address the human and financial impact of obesity and related chronic diseases like type 2 diabetes [1], [2], countries across the world are advocating the promotion of healthy lifestyles and positive lifestyle change. This is demonstrated through the publication of national guidelines, such as those which advocate for healthier diets [3] and recommend 30 minutes of moderate to vigorous physical activity on five or more days a week [4], [5]. Such guidelines are based upon evidence demonstrating the association between particular health outcomes and lifestyles such as tobacco smoking [6], [7], alcohol consumption [8], [9], physical activity [10], [11] and dietary habits [12], [13].

Some commentators have suggested, however, that the translation of this research into health policy has tended to result in ‘siloed’ strategies that attempt to modify one unhealthy lifestyle at a time [14]. Such an approach may be inefficient, since an increasing number of studies in the UK [14], [15], [16], [17], [18], Belgium [19], Finland [20], the Netherlands [21], the US [22], [23], New Zealand [24] and China [25], [26] report that unhealthy lifestyles tend to co-occur non-randomly among the same individuals. Therefore, interventions which tackle multiple unhealthy lifestyles simultaneously may be more appropriate, as has been argued in the case of diabetes prevention [27], [28].

With some notable exceptions [16], [17], [20], however, previous work which has sought to identify the social determinants of multiple unhealthy lifestyles has focused upon individual characteristics (especially socioeconomic factors) and hitherto paid little attention to the role of neighborhood characteristics (such as affluence or geographical remoteness). This is an important gap to address, since the places in which people live have long been used as targets for experiments and policy interventions[29], [30], [31], [32], [33], [34]. There is increasing widespread belief among policy makers and academics that neighborhoods can influence health outcomes independent of characteristics operating at the individual-level [35], [36], but less is known on the extent that neighborhoods also determine the co-occurrence of unhealthy lifestyles. Although individual-level factors (e.g. income) are important correlates of multiple unhealthy lifestyles, it is possible that neighborhood characteristics, such as socioeconomic circumstances, could amplify (in the case of deprived neighborhoods) or buffer (in affluent areas) the impact of those individual factors [37]. Addressing this hypothesis was the aim of this study.

## Methods

### Data

Our analyses focused on the 45 and Up Study [38]. Between 2006 and 2008, participants were randomly selected from the Medicare Australia database (the national provider of universal health insurance in Australia) and self-completed a survey on lifestyle, health status and socioeconomic circumstances. Response to the survey is estimated at 18%, though previous work has shown that the results relating to relative risks from the 45 and Up Study are similar to those from a representative population health survey [39]. All participants were resident in New South Wales (NSW), the most populous state in Australia. The University of New South Wales Human Research Ethics Committee approved The 45 and Up Study.

### Outcome variable: the ‘unhealthy lifestyle index’

Previous work has tended to construct outcome variables by summing binary indicators of unhealthy lifestyles. We took a similar approach using eight measures of unhealthy lifestyles available within the 45 and Up Study. These variables were selected based upon published national guidelines for tobacco smoking cessation [40], alcohol consumption [41], moderate to vigorous physical activity [4] and a range of dietary indicators [3]. The data and refinement of these variables was as follows:

#### 1) Tobacco smoking

Current smoking status was derived from affirmative responses to the question *“Are you a regular smoker now?”* For participants reporting a history of smoking, those who had smoked within the past year were classified as current smokers (coded as 1), whereas those who had not smoked within the last 12 months were classified as non-smokers (coded as 0).

#### 2) Alcohol consumption

Participants were asked *“how many alcoholic drinks do you have each week?” and “on how many days each week do you usually drink alcohol?”* These variables were used to identify the approximate number of alcoholic drinks consumed each day. A binary variable was constructed to distinguish between participants consuming less than (coded as 0), or at least two alcoholic drinks a day (coded as 1).

#### 3) Physical activity

The Active Australia Survey [42] was used to ascertain the number of minutes spent in moderate to vigorous physical activities (MVPA) each week. Previous work has demonstrated this survey to have a satisfactory level of test-retest reliability [43]. In line with national guidelines [4], participants 30 minutes of MVPA on five or more days a week (coded as 0) were differentiated from those who did not achieve this level of MVPA (coded as 1). Participants who met the guideline of 2.5 hours of MVPA a week, but not spread over 5 or more days, were classified as not meeting the national guideline (coded as 1) which is explicit in recommending regular, rather than concentrated participation.

#### 4) Fruit consumption

Participants responding two or more (coded as 0) to *“about how many serves of fruit do you usually have each day?”* were distinguished from those consuming less (coded as 1). Fruit juice was measured separately in the survey and not considered appropriate for this study as it would be impossible to differentiate between nutrient rich fresh juice and that from concentrate which is often high in sugar content.

#### 5) Vegetable consumption

Responses to the question *“about how many serves of vegetables do you usually eat each day?”* were counts stratified by cooked and raw varieties. We summed both responses and differentiated participants eating at least five portions of vegetables per day (coded as 0) from those eating fewer than five (coded as 1).

#### 6) Consumption of red meat and processed meat

Participants indicated the number of meat products eaten each week by the type of meat. Guidelines on consumption also differ by the type of meat. We coded participants as 1 if they ate between 3 and 5 weekly portions of red meat (beef, lamb or pork), or zero weekly portions of processed meat (bacon, sausages, salami, burgers). Participants were coded as 0 if they ate fewer than 3 or more than 5 weekly portions of red meat or 1+ portion of processed meat.

#### 7) Low-fat milk

Participants were asked to indicate which type of milk they consumed most of the time. Those who drank reduced fat milk or skim milk (coded as 0) were differentiated from other participants who either drank no milk, whole milk, or another variety (coded as 1).

#### 8) Fish

The number of portions of fish (or other seafood) eaten weekly were indicated as a count. Participants eating three or more portions of fish (coded as 0) were differentiated from those eating fewer portions per week (coded as 1).

Summing the responses of each of the aforementioned binary variables gave a score ranging from zero to eight co-occurring unhealthy lifestyles. This variable is referred to hereafter as the *‘unhealthy lifestyle index’.*

### Health status and other individual-level measures

Previous studies have demonstrated association between the co-occurrence of unhealthy lifestyles and health status [22], [23]. To perform a similar validation, we utilized three indicators available in the 45 and Up Study. General health and life quality were both self-reported and scored from 1 to 5: excellent, very good, good, fair, poor. Binary variables were constructed by aggregating responses excellent through to fair, leaving participants reporting poor health or quality of life as separate categories.

A third health variable pertained to mental health, as measured by the Kessler Psychological Distress Scale (K10). The K10 is a widely used instrument comprising 10 questions on whether a person felt tired for no reason, nervous, hopeless, restless, depressed, sad or worthless during the last four weeks. Scores for each of the 10 questions ranged from 1 (“none of the time”) to 5 (“all of the time”). When each of the scores are summed, participants with aggregate of 22 are identified as being at a high risk of psychological distress.

Other individual-level characteristics which have been previously reported as being associated with multiple unhealthy lifestyle indices were also included as control variables. These included age, gender, annual income, education qualification, economic status (**employed, unemployed, retired, inactive due to long term illness or disability**), couple status and country of birth.

### Neighborhood-level measures

To define neighborhoods, this study used Census Collection Districts (CCDs) which have a mean of 225 residents [44] and were the smallest geographical scale at which 2006 Census data was disseminated [45]. We focused on the level of affluence and geographic remoteness of neighborhood environments as widely used indicators were available. Local affluence was measured using the Socio-Economic Index for Areas (SEIFA) ‘Index of Relative Socio-Economic Advantage/Disadvantage’ [46]. This variable was initially in rank format, so it was re-expressed in percentiles; higher percentiles indicated more affluent neighborhoods. Geographical remoteness was measured using the ‘Accessibility/Remoteness Index of Australia’ (ARIA) [47]. ARIA is a score ranging from 0 to 15, with scores of 2.4 and over used to distinguish between urban and inner regions (<2.4) and rural or remote areas (> = 2.4).

### Sample

A sample of 206,457 participants with complete data on unhealthy lifestyles (smoking status, alcohol consumption, physical activity and dietary measures) and health status (self-rated health, self-rated quality of life, and risk of psychological distress) were selected from 267,151 in the 45 and Up Study. We imputed the gender-specific mean to address missing data for continuous independent variables (a ‘missing’ category was used for categorical variables). The most substantive missing outcome was for the number of minutes spent in MVPA (n = 22,136, 8.3% of the sample). Persons missing any of the outcome variables were more likely to be older and less educated, not employed or in a couple, on lower incomes and living in more deprived neighborhoods. No substantive differences in missing outcome data were found by gender or country of birth.

### Statistical analysis

The distribution of the unhealthy lifestyle index across the sample was assessed using percentages. For each of the 9 lifestyle clusters (0 to 8 inclusive), the percentage response of each individual lifestyle was calculated and graphed to examine levels of co-occurrence.

To assess the extent of correlation between the unhealthy lifestyle index and health status, multilevel binary logistic regression was used to fit associations with self-rated health, quality of life and psychological distress as outcome variables. In each of these models, the unhealthy lifestyle index was initially fitted as continuous variable, but was then substituted for a categorical version to test for non-linear relationships. Those models controlled for gender, age, education, income, economic status, couple status, country of birth, local affluence and geographical remoteness. The coefficients and 95% confidence intervals were exponentiated to odds ratios.

We then proceeded to investigate the distribution of the unhealthy lifestyle index across demographic, socioeconomic and neighborhood characteristics. The unhealthy lifestyle index was normally distributed, which afforded the application of multilevel linear regression to fit associations with each of the explanatory variables. A multilevel framework was used to disentangle associations between the unhealthy lifestyle index and factors operating at different levels of analysis; persons at level 1 nested within Census Collection Districts (neighborhoods) at level 2. The initial step in the model building strategy involved fitting a ‘null’ model (i.e. with no independent variables) to calculate the intra-class correlation coefficient (ICC). The ICC in the case of our model indicated that 1.4% of the amount of variation in the unhealthy lifestyle index could be attributed to neighborhoods. Following this, the next steps were to add in individual- and neighborhood-level characteristics sequentially, noting the magnitude and direction coefficients and to what extent they were statistically significant using 95% confidence intervals.

Interaction terms were fitted to explore for gender differences by age, and cross-level interactions between individual- and neighborhood-level characteristics (local affluence and geographic remoteness). In particular, a focus was on the potential interaction between individual- and neighborhood-level socioeconomic circumstances. Statistically significant associations were identified by using the log-likelihood ratio test (*p*<0.05). All data manipulation and analyses in this study were conducted using STATA V.12 (StataCorp, College Station, TX, USA) in 2013.

## Results

**Figure 1:**
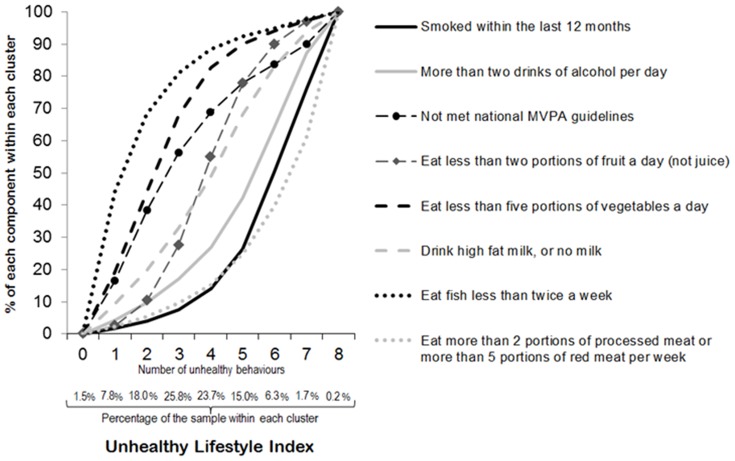
Composition of the unhealthy lifestyle index.

Figure 1 provides descriptive information on the unhealthy lifestyle index and its components. 1.5% of the sample scored zero on the unhealthy lifestyle index, whereas only 0.2% reported all eight unhealthy lifestyles. Nearly 50% of the sample reported 3 or 4 unhealthy lifestyles. The unhealthy lifestyle index followed a ‘normal’ distribution. Among people who reported up to four unhealthy lifestyles, the most common of these were not eating enough fish, followed by not meeting guidelines on vegetable consumption and moderate to vigorous physical activity. Smoking, drinking too much alcohol and a processed or red meat intensive diet only tended to be more prevalent among people who reported many other unhealthy lifestyles (i.e. scores over 4 on the unhealthy lifestyle index).

**Figure 2:**
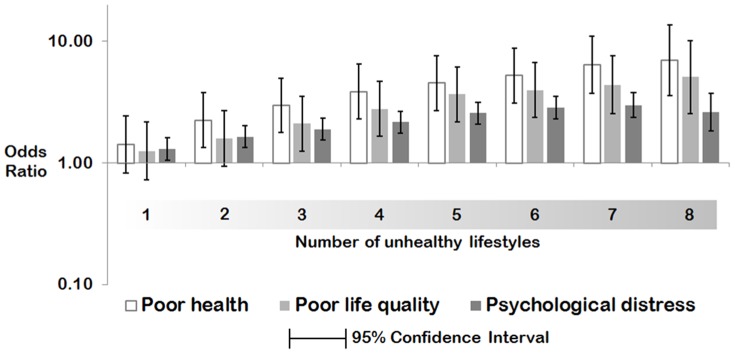
Associations between the unhealthy lifestyle index and a) poor self-rated health; b) poor self-rated quality of life; c) high risk of psychological distress (Odds Ratios and 95% Confidence Intervals are in reference to persons scoring zero unhealthy lifestyles). Models adjusted for educational qualifications, income, economic status, couple status, country of birth, neighborhood affluence and geographic remoteness.

We expected that unhealthier lifestyles would be associated with poorer health, a lower quality of life and a higher risk of psychological distress. Figure 2 confirmed these expectations. Compared to people who scored zero on the unhealthy lifestyle index (i.e. the ‘healthiest’ group), for example, those who scored 8 (the ‘unhealthiest’ group) were 7 times more likely to rate their health as poor (95%CI 3.6, 13.7), 5 times more likely to report poor quality of life (95%CI 2.6, 10.1), and had a 2.6 times greater risk of psychological distress (95%CI 1.8, 3.7).

**Figure 3:**
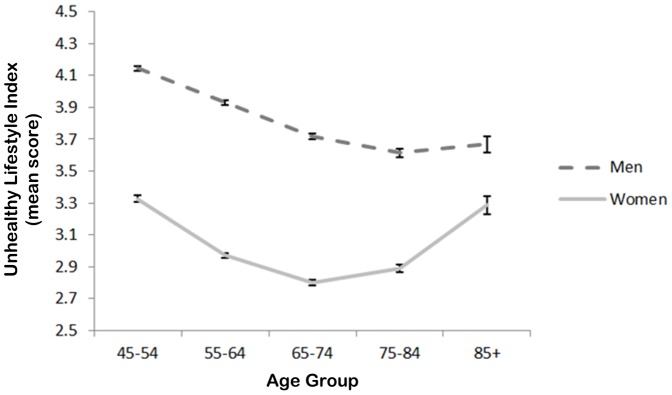
Mean scores on the unhealthy lifestyle index: interaction between gender and age, adjusted for educational qualifications, income, economic status, couple status, country of birth, neighborhood affluence and geographic remoteness.

In general, men scored higher on the unhealthy lifestyle index than women irrespective of age (Figure 3). This was despite lower (i.e. healthier) mean scores among older men. A similar improvement in lifestyle was observed for women between age 45 and 74, but from 75 onwards, the mean score on the unhealthy lifestyle index increased. The combination of decreasing scores among men and a parabolic trend for women meant the gender gap in the unhealthy lifestyle index at age 45 diminished substantially into older age.

**Figure 4:**
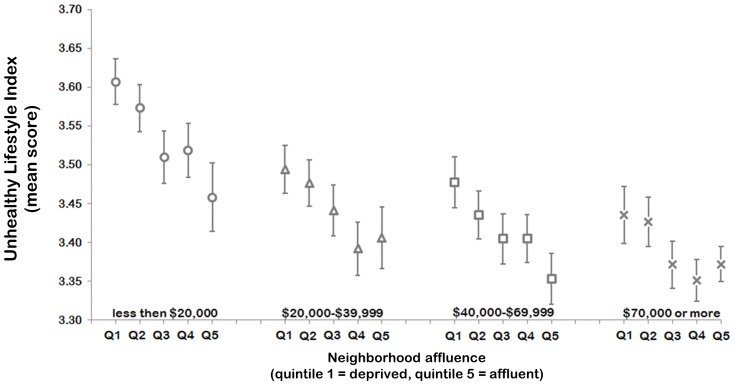
Mean scores on the unhealthy lifestyle index by annual household income and neighborhood affluence, derived from fully adjusted multilevel linear regression models. The reference group is participants earning less than $20,000 a year while resident in the poorest neighborhoods (quintile 1).

Figure 4 reports that people who lived in more affluent areas tended to score lower on the unhealthy lifestyle index, regardless of income. However, people earning less than $20,000 a year and living in the most affluent areas scored very similar on the unhealthy life index with those on higher incomes living in more deprived neighborhoods. In contrast, people with moderate incomes living in the most affluent areas scored significantly lower on the unhealthy lifestyle index than people living in deprived neighborhoods on incomes above $70,000 per year. People living in more rural and remote neighborhoods scored slightly higher on the unhealthy lifestyle index than their peers in urban areas (Coefficient: 0.02, *p* = 0.045).

## Discussion

### Main finding

In line with previous studies [22], [23], the co-occurrence of unhealthy lifestyles was associated with an increased risk of poor self-rated health, quality of life, and a high risk of psychological distress. Previous reports on the co-occurrence of unhealthy lifestyles have tended to focus on the characteristics of individuals to demonstrate social patterning [14], [15], [16], [17], [18], [19], [20], [21], [22], [23], [24], [25], [26]. The *spatial* patterning, by contrast, has received substantively less attention [16], [17], [20]. The main finding from our study was that the socioeconomic context in which people reside does have an influence on unhealthy lifestyle co-occurrence, over and above the impact of characteristics at the level of the individual. A higher income was more beneficial overall, but among people with the same level of income, the co-occurrence of unhealthy lifestyles was lower if they were resident in more affluent neighborhoods. Where a person lived appeared to matter most if their income was less than $20,000 per annum; those on low incomes in the poorest neighborhoods having highest mean number of multiple unhealthy lifestyles overall. For policy makers, this suggests that people living in poorer neighborhoods are a high risk group for the co-occurrence of unhealthy lifestyles; even if they are simultaneously on relatively high incomes. In the same vein, our findings also imply that affluent neighborhood circumstances may support healthier lifestyles even among those on low incomes. As such, future research should look to evaluate the trade-offs of investing in multiple unhealthy lifestyle interventions at the population-level versus those which target specific geographical areas, such as deprived neighborhoods.

### Interpretation

The associations between neighborhood socioeconomic circumstances and co-occurrence of unhealthy lifestyles reported in this paper using Australian data are broadly similar to previous work in Europe [16], [17], [20]. This is the first to address the issue of unhealthy lifestyle co-occurrence in Australia and the consistency of the neighborhood effects across international boundaries is reassuring. We also tested a variable which described the rurality and remoteness of the neighborhood. The slightly higher risk among those in more rural and remote circumstances relative to their counterparts in urban areas was significant, though small in comparison to that attributed to local socioeconomic circumstances. For policy makers tasked with implementing multiple lifestyle interventions, the key message based on these results is that deprived neighborhoods could be the focus of the efforts regardless of whether they are in urban or rural areas.

Previous work has reported gender and age differences in the co-occurrence of unhealthy lifestyles [15], [25], [26], but there has been no report of gender differences narrowing with age, or the suggestion of a parabolic distribution among women of 45 years and older. This is an intriguing result which could be interpreted in different ways. If we are to take these trends as reflective of lifecourse trajectories, then it is simultaneously good and bad news. It is positive that unhealthy lifestyles among men appear to decline with age, but also deeply concerning that unhealthy lifestyles co-occur more often among older women. The lifecourse trajectory explanation for this patterning of unhealthy lifestyles by gender and age can only be speculative, however, given the data are cross-sectional. It would require follow-up of this sample over time to confirm this hypothesis. In the absence of this data, it is difficult to discount other possible explanations. What appears to be a decline in unhealthy lifestyle co-occurrence among older men may be an artifact of higher mortality rates among those males who, prior to death, would have reported a fairly high co-occurrence of unhealthy lifestyles. To test this hypothesis, longitudinal data with linked mortality records would be required. Nevertheless, the survival hypothesis would not appear to have a strong *prima facie* case to explain the parabolic trend among women. A third possibility, therefore, is potential for cohort effects, in which these age and gender differences are the product of early life experiences for people growing up in different periods of time. This may result in systematic differences in the co-occurrence of unhealthy lifestyles according to (unmeasured) variables which are correlated with age, such as health literacy [48]. Unfortunately, no data on health literacy was available in the 45 and Up Study to test this hypothesis. The patterning of unhealthy lifestyle co-occurrence by age and gender among middle-to-older age adults is, therefore, an important avenue for further exploration with longitudinal and linked data that is beyond the scope of this cross-sectional study.

### Strengths and limitations

The emphasis on people aged 45 and older was inherent within our study design, which means that our results are unlikely to reflect the situations of those under 45 years old. Although this is a limitation, the focus on middle-to-older age is under-researched in the context of investigations into multiple unhealthy lifestyles and can therefore also be interpreted as a strength. Further strengths include the large sample size and also the number of lifestyle measures, which included the consumption of fish, milk, red and processed meat, which tend not to be included in other studies that focus only on smoking status, physical activity, the consumption of alcohol and intake of fruit and vegetables. Although the 45 and Up Study was not designed to be nationally representative, previous work has demonstrated that relative risk estimates are broadly comparable to a representative population survey [39]. While ethnic differences in health [49] and lifestyle [50] are known, our study did not explicitly investigate to what extent unhealthy lifestyles clustered by ethnicity. By definition, the study also did not explore variations between Indigenous and non-Indigenous Australians and how these play out spatially; this marks another avenue for future exploration. Another area for further investigation is the question of what it is about deprived neighborhoods that increases the risk of unhealthy lifestyle clustering, such as a potential lack of access to green spaces [51], [52] or other opportunity structures.[37] Finally, a reliance on cross-sectional data prohibits causal inference, though longitudinal analyses will be possible to test the robustness of the associations in this paper when the follow-up wave of the 45 and Up Study becomes available.

## Conclusion

Previous work on the determinants of co-occurrence in unhealthy lifestyles has tended to focus mainly upon the characteristics of individuals. The results of this study suggest that the socioeconomic circumstances of where a person lives could have an impact on lifestyle co-occurrence which is independent of their individual characteristics. Where a person lives appears to have a more substantial influence if they are on a low income, yet even people on higher incomes tend to have unhealthier lifestyles if they also live in poor neighborhoods. The key message for policy makers is that unhealthy lifestyles co-occur more strongly among residents of deprived neighborhoods, regardless of their individual demographic and socioeconomic circumstances. Future research, therefore, should investigate the trade-offs of a population-level approach towards intervening on multiple unhealthy lifestyles versus one which targets resources towards specific geographical areas.
